# Lancing the Gums

**Published:** 1875-03

**Authors:** James Finlayson


### ARTICLE VII.

### *Lancing the Gums.*

Dr. James Finlayson, in a very elaborate and learned paper on the Dangers of Dentition (*Obstetrical Journal of Great Britain,*) Dec., 1873, Jan. and Feb., 1874,) states that the tendency of opinion at present seems to assent to Dr. West's dictum, that "the circumstances in which the use of the gum lancet is really indicated are comparatively few." Rilliet and Barthez could only recall one case in which any real benefit resulted from the operation, and the best Trosseau could say of it was that the practice was useless. Even the most skeptical, however seem to have encountered rare cases where convulsions ceased on the lancing of the gums; such results are also obtained at times from other most unlikely remedies. It may here be stated that in his careful study of 102 cases of infantile convulsions, Dr. Gee could find no reason to believe that teething bore any part in the causation of the fits, and in none of the cases did it seem necessary to lance the gums.

But it may be said, although the benefit may be very doubtful, why hesitate to give any child the chance of profiting in its peril or suffering by such a simple operation? It is very probable that this idea regulates the conduct of many in dealing with infantile disorders. Such a proceeding has very properly been stigmatized as "nothing better than a piece of barbarous empiricism, which causes the infant much pain, and is useless or mischievous in a dozen instances for one in which it affords relief." It may, however, be well to consider shortly whether the absence of danger from lancing is so complete as is usually represented. And here we may call in evidence the great modern upholder of the practice—Marshall Hall—himself. He was much too consistent an advocate of his own views to ignore the danger of such frequent tampering with the mouth and gums of an excitable infant as he had himself recommended, and he admitted this disturbance as a real and true objection to the use of the gum lancet. Such a course of treatment is indeed well calculated (as an American physician says) to "make your child your mortal foe." But this objection—no trivial one when fully considered—is not all. Local disasters have also happened. Passing by as doubtful any injurious influence on the ultimate growth of the teeth, suppuration and ulceration of the gums, and even gangrene, are admitted by its advocates to have been seen after this operation. Dangerous or fatal hemorrhage from lancing the gums, although not like to be readily recorded, has been published in several cases. Even M. Baumes admits the danger from hemorrhage in incising the gums when much engorged; and he points out that the swallowing of the blood may conceal the extreme peril of the infant. Hamilton, although he had never seen a death from this cause, heard of one on evidence which he could not controvert. Dr. Churchill admits that bleeding from the wound has sometimes been excessive, requiring pressure, astringents, and caustics. Rilliet and Barthez have known it to require plugging. Dr. B. W. Richardson speaks of having

"had two or three very painful lessons of this description," and mentions one death occurring to a country practitioner, and another accident with nearly fatal syncope in his own dispensary practice. Dr. Young, of Edinburgh, narrated a few years ago two deaths which occurred in his father's practice. Fatal hemorrhages have also been reported by Taynton, Anderson, Whitworth, Des Forges, and Nicol, and in only one of these cases was there supposed to be any special hemorrhagic tendency. Further scrutiny of these cases show, as we might expect, that nearly all the deaths were reported under exceptional circumstances, so that many more disasters have doubtless occurred, and have been allowed to slip into oblivion. Without laying undue stress on these perils and calamities, occurring as they do amongst such an enormous number of operations, they may well be seriously considered *when the generalization of the treatment is contended for on the ground of its absolutely innocuous character.—Medical News and Library..*

---

